# Reviews and Book Notices

**Published:** 1883-05

**Authors:** 


### Reviews and Book Notices.

ARTICLE VII.—A PRACTICAL TREATISE ON DISEASES OF THE SKIN. By JAMES NEVINS HYDE, A.M., M.D. Philadelphia: Henry C. Lea's Son & Co. 1883.

The earliest record of any skin disease that we can find seems to be in the description of leprosy in the Old Testament, Leviticus 13 and 14, where the disease is described with some minuteness as to its characteristics and diagnosis, with instructions as to the purification and separation of those afflicted, though the cure was apparently as impossible, in those early times, as at the present.

In the writings of Hippocrates, also, dating back at least twenty-two hundred years, may be found descriptions of several forms of skin diseases, described with accuracy, and, according to the accounts, successfully treated.

From those old days to the present, to what varieties of treatment have these diseases been subjected, influenced by the thousand and one ever varying theories of the cause of disease, the solidism of one century and the humoralism of another!

Within the last thirty years, however, the advance in the study and treatment of skin diseases has been most marked, fully keeping up with what has been done in other departments of medical science. Without wishing to depreciate what has been done by the English and French schools, it is safe to say that the profession is indebted to Hebra, of Vienna, more than to any one else, for bringing our knowledge up to some definiteness and certainty. Beginning, like a true German skeptic, by not accepting any of the older theories and practice till he had subjected them to the test of his own experience, he began by investigations

*de novo*, and from the almost unlimited amount of material at his disposal in the hospitals of Vienna, brought the science of dermatology to a higher plane than it had ever reached before. Thousands of pupils have been attracted to Vienna, to sit at the feet of the great master, and the strong impetus given to the study of skin diseases has shown itself in the increased number of observers all over the world.

It is to give to the profession at large the benefit of what these workers have accomplished, and to show what has been done up to the present time, that this work has been undertaken, and most admirably has it accomplished its object. In this book the practitioner, busy or otherwise, may find what we really know about skin diseases up to this year of our Lord 1883, and withal submitted to the judgment of a man of study and experience in this department of medical science.

While there is no branch of medical study which is so impossible to learn from books alone as dermatology, yet the student who wishes to correct and confirm his observations of skin diseases; to supplement and arrange what he has learned from the actual observation of cases, will find no better guide than the present volume.

If we were to instance any chapters which seem to us particularly good, we should mention that on eczema. In that, as, in fact, all through the book, is taught the great lesson, to which sufficient prominence has never, we think, been given in works on general or special practice, that the successful practitioner must treat, not the disease, as a disease, but the patient as he stands before him. He must prescribe for the wants of just that particular individual who consults him, taking into consideration what his ancestors gave him, and what his present condition is as to habits, employments, and all his surroundings.

This same remark would apply to the chapter on acne, a skin disease, in the proper treatment of which the skin is about the last thing to take into consideration. Acne is a disease of defective innervation, and whatever has produced this influence on the nerves is the main thing to be treated, all other treatment being only slightly adjuvant.

In the chapters on syphilis will be found a good *résumé* of the existing state of knowledge on this interesting subject, and the directions for treatment are judicious and comprehensive. The importance of attending to the general health ; of out-door exercise; generous diet; iron and proper tonics, as well as the routine mercurial treatment, is very properly insisted on.

On the whole, the work seems to us to be a clear and concise statement of what the profession wants to know, and it will prove a most valuable guide to the thousand students who annually go out from Chicago, to make their professional career in the great Northwest. C. G. S.

ARTICLE VIII. CONTRIBUTIONS TO ORTHOPEDIC SURGERY. Including Observations on the Treatment of the Hip, Knee and Ankle Joints by a New and Simple Method of Extension. And Lectures on Club Foot, by Jas. C. Hutchinson, M. D. New York. G. P. Putnam's Sons.

The author states at the outset that all the morbid conditions of the joints are, as a rule, essentially chronic, and characterizes attempts to describe the symptoms indicating distinct pathological states of the individual structures of the joints as mere pathological refinement. He abandons all apparatus in coxalgia except the crutch and elevated shoe on the unaffected side. Extension is secured by the weight of the limb. He maintains that sufficient immobility is secured by the reflex contraction of the periarticular muscles.

In the treatment of knee-joint disease, the author uses the same plan with the addition of compression by a splint consisting of seven layers of shellaced muslin, which is laid upon the knee while extended.

In the diseased ankle joint a plaster splint is recommended, applied during extension, and renewed from time to time while using the elevated shoe and crutches. The author claims this treatment to possess advantages over that commonly employed in the management of chronic inflammation of the joints of the lower extremities. In that the surgeon is saved the trouble and annoyance of applying and carefully watching the instruments in ordinary use to see that proper extension is kept up, and undue pressure prevented, while the patient's comfort is greatly promoted by dispensing with adhesive plasters, which irritate the skin, and require removal from time to time, and by discarding the perineal band in hip disease. The spasmodic contraction of the periarticular muscles is overcome by the gentle physiological extension produced by the weight of the limb for several hours each day, while forcible extension by weight and pulley irritates the muscles to resistance and contraction, which must be overcome by main force.

The inexpensiveness of the apparatus, which is easy of construction by any ordinary mechanic, is a matter of no small importance.

In the five succeeding lectures on club foot, the author, after a short historical sketch of the rise and progress of orthopedic surgery, takes up each of the four varieties of talipes and their four compound forms in anatomical and surgical detail.

While there is nothing particularly new, yet all the information necessary to the subject is developed in attractive and compact form. W. L. D.

ARTICLE IX.—VARIOLA. A series of Twenty-one Heliotype Plates, Illustrating the Progressive Stages of the Eruption. Boston: Samuel A. Powers, 1882. Price $3.00

This is a book of beautiful plates, made and arranged under the direction of Mr. Samuel A. Powers, Superintendent of the Small-pox Hospital, Boston.

The plates represent the appearance of Variola at the different stages of the eruption. The first sixteen pictures show its appearance on the same patient from the third to the fourteenth day, when it began to disappear. The remaining six pictures were taken from different patients in same hospital to show some special feature in the disease.

The book will help out the work of diagnosis in many cases.

Mr. David F. Hicks, of Hyde Park, Ills., is the agent for its sale. D. R. B.

ARTICLE X.—A TREATISE ON DIPHTHERIA AND CROUP. By Charles J. Lewis, M. D., Alumnus of Rush Medical College, etc., Chicago. Published by Clark & Edwards, 1883. Price $1.00.

This is an admirable little monograph, full of practical suggestion for the management of these formidable diseases, according to the author's pathology, best expressed by the following extracts from his preface: "Having felt for several years the pressing need of establishing a close relation between a generally successful treatment of diphtheria and croup with antiphlogistics and eliminants, and a theory of the nature of these diseases that was consistent with such management, is my plea for bringing out this treatise.

"M. Bretonneau regarded diphtheria as an inflammatory disease, and treated it as such. His views of the essential nature of the disease were correct; but his methods of cure are so crude, so inadequate, in not giving due prominence to eliminants, that his antiphlogistic treatment was, before his death, abandoned as a failure. This failure would not have been attained had he selected anti-inflammatory and eliminatory drugs, with due regard to their physical action. I have given due prominence to the action of both these classes of medicines. I am not conscious of a diphtheria and croup poison—a contagium vivum—hence, the claim of contagion and of germ origin, is unproven."

The method of treatment proposed is ingenious, plausible, and we have reason to believe, has been, in the Doctor's hands, very successful.

We take pleasure in commending the book to the favorable attention of physicians. D. R. B.

---

THE will of Sir Thomas Watson has been proved. The personalty alone amounts to more than £164,000. A modest fortune for a life of distinguished professional services.

